# Direct Observation of Palladium Leaching from Pd/C by a Simple Method: X-ray Absorption Spectroscopy of Heterogeneous Mixtures

**DOI:** 10.1021/acsomega.3c01343

**Published:** 2023-06-07

**Authors:** Kenichi Uno, Takanori Itoh, Hidenori Sato, Guy C. Lloyd-Jones, Yuya Orito

**Affiliations:** † Process Technology Research Laboratories, Daiichi Sankyo Co., Ltd., 1-12-1 Shinomiya, Hiratsuka, Kanagawa 254-0014, Japan; ‡ Physical Properties & Chemical Analysis Department, NISSAN ARC, LTD., 1, Natsushima-cho, Yokosuka, Kanagawa 237-0061, Japan; § School of Chemistry, The University of Edinburgh, Joseph Black Building, David Brewster Road, Edinburgh EH9 1FJ, U.K.

## Abstract

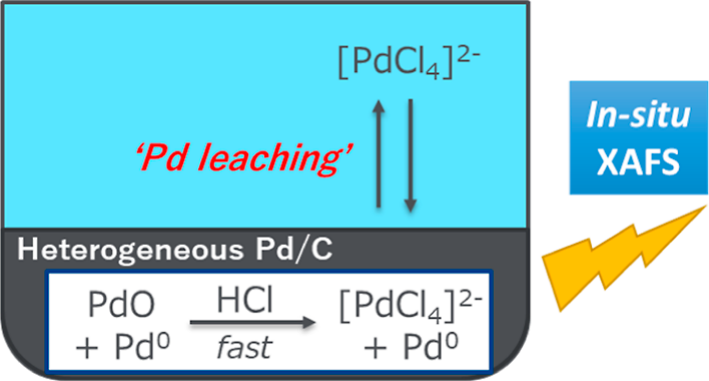

Herein, we show the detailed behavior of palladium leaching from palladium on charcoal by aqueous HCl, directly observed by X-ray absorption spectroscopy measurement employing a simplified reaction setup. While Pd^0^ is not affected by the addition of HCl, palladium oxide in nanoparticles readily reacts with HCl to form the ionic species [Pd^II^Cl_4_]^2–^, even though these ions mostly remain adsorbed on the surface of activated charcoal and can only be detected at a low level in the solution phase. This finding provides a new aspect for control of the leaching behavior and robust usage of palladium on charcoal in organic reactions.

## Introduction

Palladium on charcoal (Pd/C) is widely used in the field of synthetic organic chemistry, from the laboratory scale through to industrial plants.^1−4^ The immobilization of Pd nanoparticle catalysts on the surface of activated charcoal, has many benefits in use, e.g., easy handling, storage, and removal, together with its generally high catalytic activity.^5,6^ In the utilization of this catalyst, the matching of the type of Pd/C and the target reaction is known to be important, along with an appropriate selection of reaction conditions such as acidity, solvent, pre-reduction, etc.^7,8^ On the other hand, the behavior and role of Pd varies depending on the type of reaction. For example, hydrogenolysis proceeds as contact hydrogenation with adsorbed hydrogen molecules,^1^ in contrast to cross-coupling in which Pd is leached into the solution phase to form an active organometallic complex.^9−17^ In the application to commercial production processes, the leaching of Pd into the solution phase is quite commonly observed, albeit usually in ppm quantities, and needs to be controlled by regulation in line with ICH guidelines of drug substances.^18^ Thus, predictive control of the behavior of Pd/C is a key aspect to the construction of robust chemical processes, but the roles and mechanisms of well-known techniques like pre-reduction or pH control are not fully understood, especially at the nano-scale. Herein, we report on the detailed analysis of the reaction profile of Pd/C with HCl in slurry using transmission XAS (X-ray absorption spectroscopy) as a simple and direct new method for observation of the Pd species.

XAS is an X-ray analysis method that provides direct information about chemical species, oxidation states, and nano-scale structures around a targeted atom by analyzing the fine structure of the spectra (also known as XAFS; X-ray absorption fine structure).^19^ To achieve high spectral resolution, a high-energy synchrotron emission beam is used for the measurement. Using X-rays, which can pass thorough materials which have lower interaction such as reaction vessels, solvents, and activated charcoal, appeared to be an ideal method for the direct analysis of palladium speciation on charcoal. Recently, applications of XAS analysis have expanded from focusing purely on inorganic species to the investigation of the solution-phase structure of organometallic catalysts.^20−26^ Nevertheless, the application of XAS to reaction monitoring is still rather rare due to the technical limitations associated with sample preparation.^27,28^ While the XAS transmission method normally takes at least 1 min per scan,^29^ the solution concentrations of catalytic organic reactions are usually too low to be measured by this method. Fluorescence mode of XAS is thus the preferred method for analysis of organic reaction solutions, although it requires a much longer scan time, ranging from 15 min to several hours. At the starting point of this research about heterogeneous Pd/C slurry, we decided to re-investigate and to challenge this limitation of XAS transmission to develop a direct analytical method for batch organic reactions.

## Materials and Methods

### XAS Instruments Setup

XAS measurements were carried out using PF-AR NW10A beamline of the Photon Factory (PF) in the High Energy Accelerator Research Organization (KEK, Tsukuba, Japan). The storage ring was operated at 6.5 GeV. The white light was monochromatized using a water-cooled double crystal monochromator equipped with Si(311) crystal. The XAS spectra of the Pd K-edge (24.35 keV) were recorded in transmission and fluorescence modes. The X-ray beam size was 2 mm (width) × 1 mm (height) by using a four-quadrant slit. The incident X-ray beam intensity (*I*_0_) were monitored by a 170 mm long ionization chamber with Ar gas 100%.

### Materials

Pd/C was purchased from Kawaken Fine Chemicals (Type M, 5 wt %, ca. 50% wet) and stored under an ambient atmosphere unless otherwise noted. Water was processed with Millipore Milli-Q. Hydrochloric acid and other acids were purchased from Wako Pure Chemical and used as received. Reference [Pd^II^Cl_4_]^2–^ solution was prepared from Na_2_PdCl_4_ purchased from Sigma-Aldrich. For PdCl_2_, in-house standard in KEK was used as prepared. Spectrophotometry cells and plastic reaction vessels were purchased from As One corporation.

See the Supporting Information for full experimental details.

## Results and Discussion

### Development of Transmission XAS Conditions

In a typical transmission XAS experiment, samples are prepared as a pressed pellet with a short optical path, typically 1–3 mm.^30^ In terms of concentration, 1–5 wt % Pd is needed to obtain a good S/N ratio in the measurement. However, the palladium concentration in a typical organic reaction is generally far lower than this, even in reactions conducted at what would be considered “high catalyst loadings”. For example, the Pd concentration at 5 mol % in a 0.5 M reactant solution is only 0.26 wt %. To achieve palladium concentration appropriate for XAS, it would require either a 5 M reactant solution, or a 50 mol % catalyst loading, neither of which are likely to be representative of the actual reaction conditions being employed in the process. On the other hand, since transmission XAS operates by fundamentally the same principles as optical absorbance spectroscopy, extending the optical path length should enhance the S/N ratio. In short, using a 10 cm pathlength is expected to provide an increase in S/N equivalent to up to 2 orders of magnitude higher concentration of reaction solution at the normal pathlength. Based on this aspect, the increase in background signal from the absorption and elastic scattering by solvent and vessel molecules are expected to be the main cause of erosion of signal quality. However, in the case of hard X-ray analysis at the Pd K-edge, interactions of X-ray between the light elements present in organic compounds are expected to be relatively low. We thus decided to investigate the vessel materials and solvents which provide a minimal rise in the background signal at longer optical path measurement of transmission XAS.

To quantify the background signal raise caused by the absorption of vessel material and solvent molecules, the raw absorption count (μt) was evaluated without normalization. The energy gap (Δμt) should be >0.5 to obtain satisfactory S/N ratio for processing, and the detector instruments are saturated when μt > 3, so the target background signal level was set to be μt < 2.

**Figure 1:**
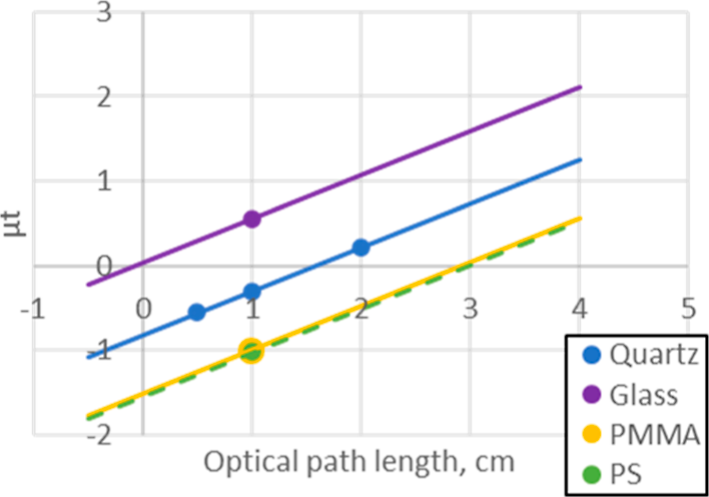
Background evaluation using water in photometer cells. Dots are measured data and lines are assuming same slopes as quartz. *Y*-intercepts give the blank absorption values of each materials.

Initially, we tested the vessel materials using commercially available sample cells for an absorption photometer. These cells are produced with good accuracy in terms of wall thickness and optical path length. To clarify the difference of elemental composition, cells made of normal soda glass, quartz glass, polystyrene (PS), and polymethyl methacrylate (PMMA) were selected. The cells were filled by water; then, the X-ray beam passed through it and the background signals recorded. First, quartz cells with multiple lengths (0.5, 1, and 2 cm) were evaluated to determine the absolute values of the background, and the linearity of absorption of water, Figure 1.

Extending the optical path length to 2 cm resulted in a satisfactorily low background signal, and a good linearity of response (blue line, Figure 1). In this case, the *Y*-intercept obtained from linear regression corresponds to the absorption of the blank sample cells. In terms of the type of cell material, normal soda glass cell showed a high background, whereas both plastic-based cells showed lower background, as expected. The difference in behavior of the vessel material mainly arises from the presence of elements such as Si, Na, etc. which are heavier than the main component of plastic vessels or solvent, such as C, O, and F. Based on this result, plastic reaction vessels are expected to be applicable for use with longer optical paths for efficient transmission XAS measurements.

**Figure 2:**
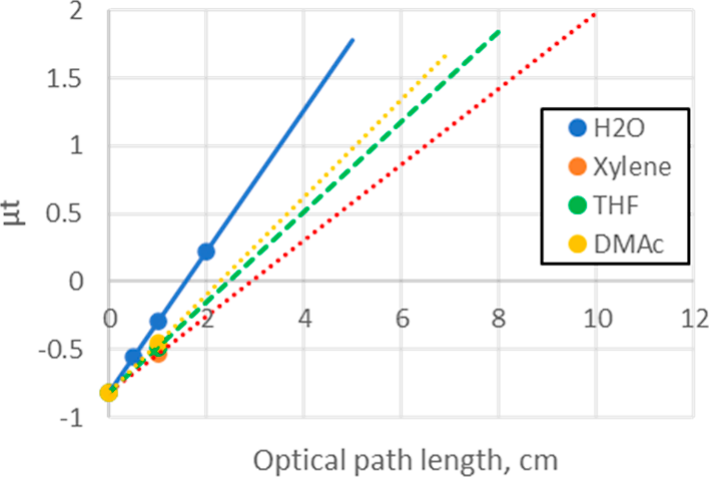
Background evaluation using organic solvents in 1 cm length quartz photometer cell. Lines are extrapolation assuming linearity of absorption.

Next, the effect of the solvent was evaluated, Figure 2. Using a 1 cm quartz cell, the background absorptions of selected organic solvents were measured and compared with water. The intensity of the background absorption increased in the order xylene < DMAc < THF < water, consistent with the concentration of oxygen atoms which have a relatively high X-ray interaction. Since the wall thickness of the quartz vessel is the same, the absorption by the vessel material is constant. So, assuming a linear response, in organic solvents, the optical pathlength in the range of up to 5–10 cm are possible (Figure 2: dashed lines).

**Figure 3:**
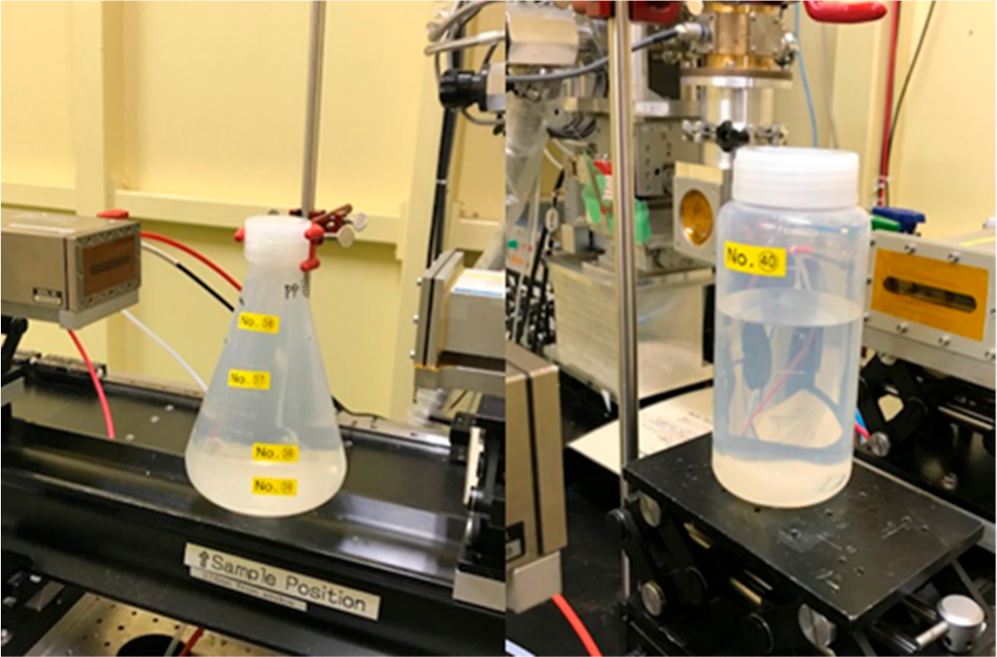
Examples of the PFA reaction vessel.

Since these spectrophotometry cells are not suitable vessels within which to perform organic reactions, we employed simple flasks constructed from polypropylene (PP) and perfluoroalkoxy alkane (PFA) polymers. These reaction vessels are applicable to both organic reactions and transmission XAS, Figure 3. The PP and PFA materials only consist of light (second period) elements and are therefore expected to have high X-ray transparency together with good chemical stability. Furthermore, when using round-bottomed or conical shaped vessel, the optical path length can be modulated simply by choosing the position and direction of the X-ray beam. Using this method, the background absorption level with long optical pathlengths were then evaluated to test the predictions based on the earlier experiments in small cells.

**Figure 4:**
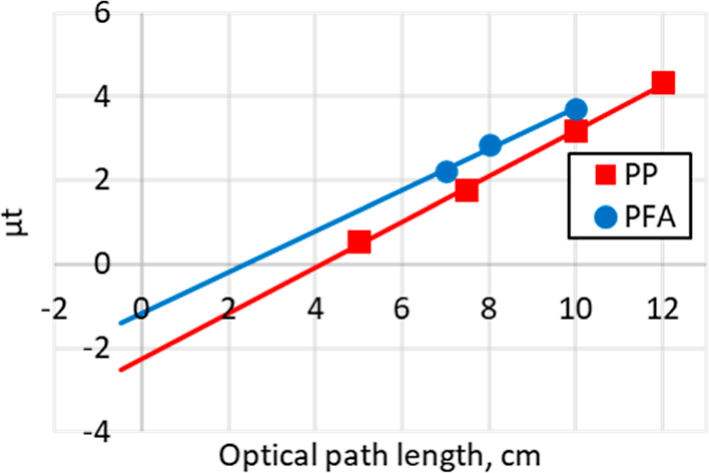
Background level of water in plastic vessels for various optical pathlengths.

The PP and PFA reaction vessels were filled with pure water and the background absorption values were recorded, Figure 4. Various optical path lengths were evaluated for conical and round-bottomed flasks, and cylindrical bottles. Both materials, i.e., PP and PFA, showed good linearity up to μt > 3, in other words, the solvent effect on the background absorption across long optical pathlengths was proven to be predictable and satisfactorily low. The vessel made of PFA gave a slightly higher background than PP, possibly due to fluorine atoms in PFA which have higher absorption of X-ray, while PP contains only C, H, and O. Using the same conditions, the whole scan range (24,000–25,900 eV) of the Pd K edge was measured to confirm that the background absorption is satisfactorily flat in the targeted scan area, see the Supporting Information for full details.

**Figure 5:**
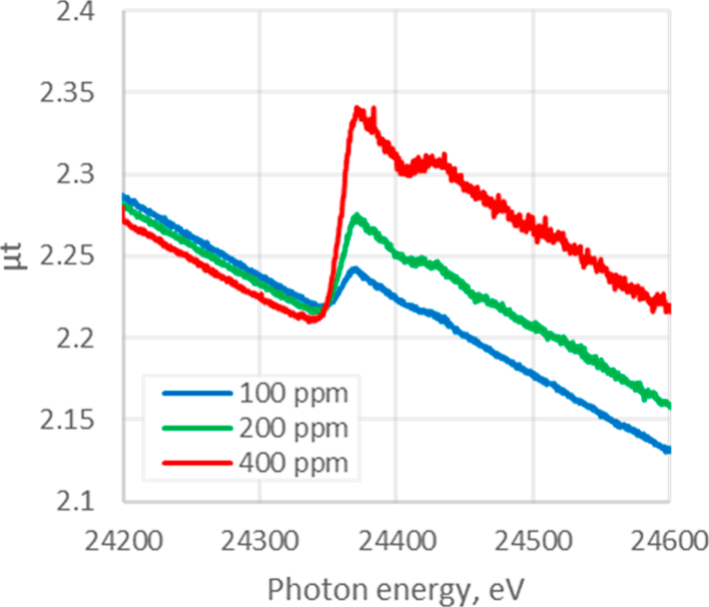
XANES spectra of Pd/C slurry in water, at the overall Pd concentrations indicated, in a PFA bottle with a 7 cm optical path length.

With the new simple long optical path transmission XAS in hand, the absorption of Pd/C slurry in water was measured using a cylindrical PFA bottle. A concentration-dependent absorbance by Pd was clearly observed, and at acceptable S/N levels, even for low Pd concentrations of 100–400 ppm, Figure 5.

### Direct Reaction Monitoring of Pd/C and HCl

**Figure 6:**
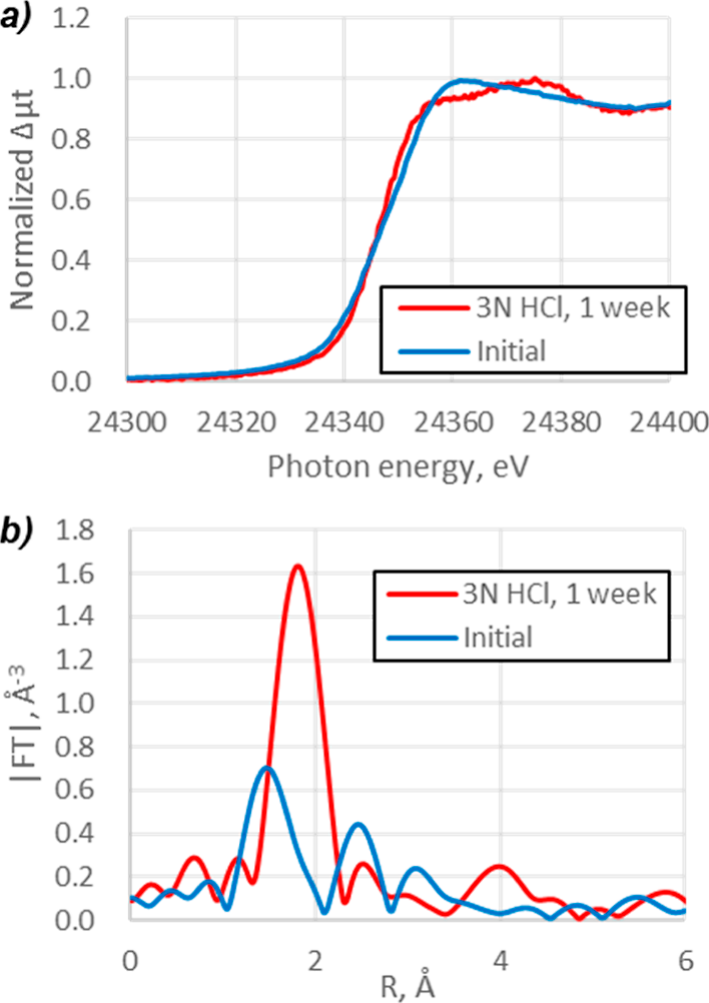
(a) XANES and (b) EXAFS spectra of Pd/C slurry in 3 N HCl obtained by the fluorescence mode of XAS.

To start the investigation into palladium leaching, Pd/C was simply mixed with 3 N HCl for 1 week and the slurry was measured by fluorescence mode of XAS, Figure 6. The treatment with HCl resulted in complete chemical respeciation of the palladium, with the XANES and EXAFS peak shapes similar to the signal of reference sample of PdCl_2_ (see the Supporting Information). Furthermore, when other acids were tested using the same method, the Pd again underwent full chemical respeciation, irrespective of the acid strength, see the Supporting Information for full details.

**Figure 7:**
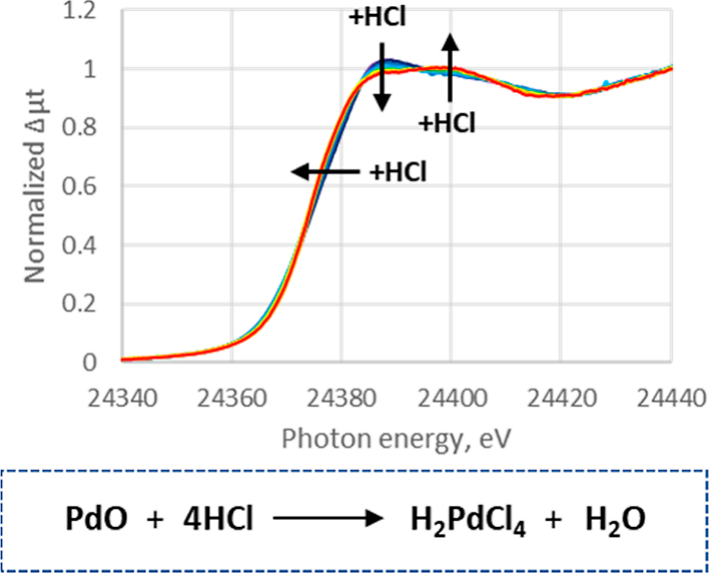
XANES spectra change along with HCl slow addition. Initial: blue and end: red.

Next, the reaction of Pd/C with HCl under slow addition conditions was monitored by the new transmission XAS transmission method, with scans at 2 min intervals. Concentrated HCl was added dropwise, using a syringe pump, to a slurry of Pd/C in water in a PP bottle, until the HCl concentration reached 0.5 M. As shown in Figure 7, the XANES spectra changed depending on the amount of HCl that has been added, and a linear combination analysis (LCA) of the XANES signal of resulting mixture showed the composition of Pd species for ca. 3% Pd^0^ and 97% [Pd^II^Cl_4_]^2–^ (less accurate due to narrowed scan range to shorten scan cycle time; see the Supporting Information for details), whereas initial composition was ca. 3% Pd^0^ and 97% Pd^II^O.^31−35^ This suggests that Pd^0^ remained intact under this condition, in stark contrast to Pd^II^O which fully reacted with the HCl. When the Pd/C was pre-reduced by saturating with H_2_ gas (1 atm.) for >1 h before experiment, no change was observed in the XANES spectra.

**Figure 8:**
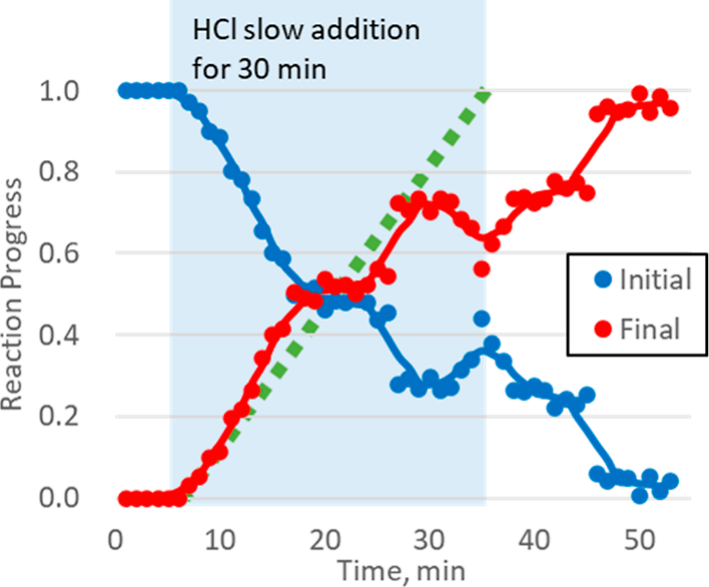
HCl slow addition reaction time course. The reaction progress was calculated by LCA of XANES of initial and final mixtures. Lines are showing 5 point moving average. Green squares indicate HCl addition.

**Figure 9:**
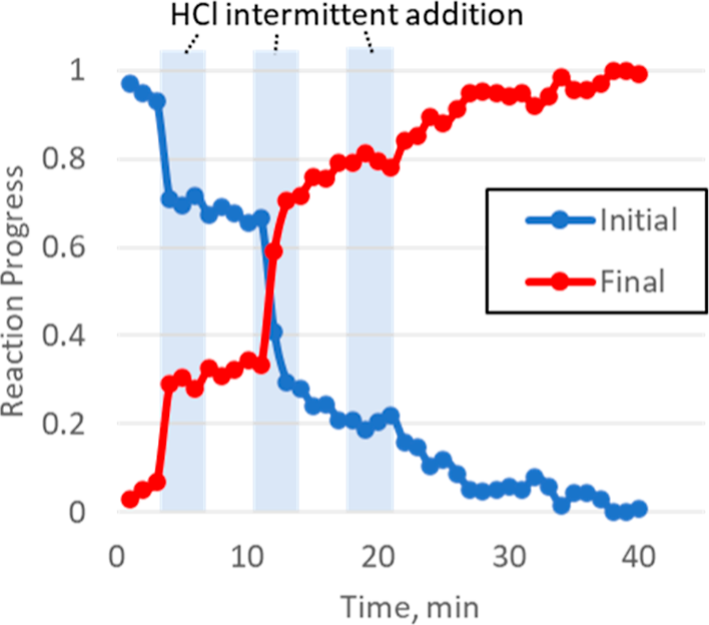
HCl intermittent addition reaction time course. Reaction progress was calculated by LCA of XANES of initial and final mixtures.

To investigate this process in further detail, the reaction progress with HCl was analyzed by LCA against time. Since linear combination fitting of three components (i.e., Pd^0^, Pd^II^O, and [Pd^II^Cl_4_]^2–^ in this case) is far less reliable than that of two due to the risk of overfitting and may results ambiguity on analysis, the LCA was performed between the spectra obtained from the initial and the final mixture.^36^ As shown in Figure 8, up to the addition of about 1 equiv of the HCl the Pd^II^O reacts immediately leading to 0.5 reaction progress at this stage. After this point, the reaction rate decreases considerably, and becomes non-linear with respect to the HCl addition rate. The reason for this behavior is unclear, but it may arise from reactivity differences between Pd nanoparticles of different diameters.^7,29,32,37^ The same behavior was observed when the 1 equiv of HCl was added intermittently in three portions, with each addition taking approximately 3 min duration, Figure 9. The change in the Pd composition was rapid during the addition of the first two portions, with the final portion resulting in no immediate response. Overall, these results suggest that the reaction between Pd^II^O and HCl is rapid, with the majority of the Pd^II^O in the Pd/C slurry being converted to ionic species within just a few minutes.

**Figure 10:**
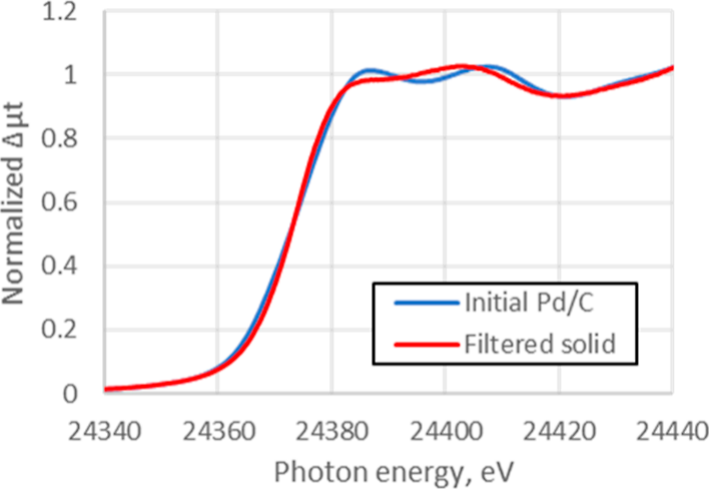
XANES spectra change of Pd/C by acid treatment. Blue: initial, red: after treatment with 1 M HCl for 0.5 h (filtered solid).

To further explore the process, we analyzed the extent of Pd leaching into solution using standard laboratory-based methods. When the Pd/C (Pd^0^ 23%, Pd^II^O 77%; determined by LCA) was stirred in 1 M HCl for 0.5 h and filtered, the amount of Pd detected in the solution phase was only 3% of total Pd mass based on Zeeman atomic absorption spectroscopy (AAS) measurements. To investigate this difference, the Pd/C, containing 97% of its initial palladium loading, was collected, washed with water, and analyzed by XAS. The spectra indicated that almost all of the Pd^II^O had been converted into [Pd^II^Cl_4_]^2–^ (Pd^0^ 24%, [Pd^II^Cl_4_]^2–^ 76%), Figure 10, likewise the result observed in the slurry, which is discussed above in Figure 6. This result means that the majority of the ionic Pd was not released into solution, but remained adsorbed on the surface of the charcoal, even though the Pd^II^O had been rapidly and near-completely converted into [PdCl_4_]^2–^, as discussed above.^38^ Moreover, the amount of Pd that was leached into solution was found to be temperature-dependent, see the Supporting Information for preliminary results. Thus, the dissolution may be controlled by an adsorption equilibrium on charcoal, although further investigation is needed to establish the details of this.

## Conclusions

In conclusion, we have shown that the reaction of Pd nanoparticles on Pd/C with HCl can be studied in detail using the simple and readily applied method of XAS. We have found that the Pd^II^O in the particle readily reacts with HCl to afford [PdCl_4_]^2–^, and that despite it mainly being only adsorbed on the surface of charcoal, only a small proportion is released (“leached”) into aqueous solution. This finding suggests that the observable Pd leaching, which is usually measured indirectly by Zeeman AAS or IPC-MS etc. is only a small proportion of the reacted Pd. Moreover, as the extent of leaching is controlled by adsorption of ionic Pd species onto the activated charcoal, this suggests that the introduction of hydrogen gas into a mixture containing ionic species may cause Pd reprecipitation and/or changes in the particle size. As this will affect the catalytic activity of the Pd/C, pre-reduction may play an important role in obtaining good reproducibility in Pd/C catalyzed reactions. In other words, by using H_2_, or other reductant, to convert Pd^II^O into Pd^0^ on the surface of the Pd nanoparticle, one may efficiently prevent subsequent reactions with acid. Further investigations to reveal the full behavior of the Pd/C leaching phenomenon and its effects on catalytic activity are underway. We also propose that the long optical pathlength transmission XAS described herein offers the possibility for application in the direct measurement of organic reactions.

## Supporting Information Available

Detailed information for in situ XAS experiment setup, spectra, HCl intermittent addition, LCA for in situ reaction monitoring, background level of the whole scan area, initial investigation of the reaction of Pd/C with HCl, reactions of Pd/C with other acids, reaction of Pd/C with HCl and analysis of liquid and solid phase after separation, and temperature dependency of the amount of Pd leaching into solution (PDF)
XAS data (ZIP)

## Abbreviations

PP: polypropylene
PMMA: polymethyl methacrylate
