# Supplementary material for: Direct Observation of Palladium Leaching from Pd/C by a Simple Method: X-ray Absorption Spectroscopy of Heterogeneous Mixtures
Source: ACS Omega. 2023 Jun 7;8(24):21787–92. doi: 10.1021/acsomega.3c01343 (PMC10286095; doi:10.1021/acsomega.3c01343)
Supplement: Supplementary file 1 — ao3c01343_si_001.pdf [file ao3c01343_si_001.pdf]

Supporting information

Direct observation of palladium leaching from Pd/C by a simple method: X-ray absorption spectroscopy of heterogeneous mixtures

Kenichi Uno[a], Takanori Itoh[b], Hidenori Sato[b], Guy C. Lloyd-Jones[c], and Yuya Orito[a]*

[a] *Process Technology Research Laboratories, Daiichi Sankyo Co., Ltd. 1-12-1 Shinomiya, Hiratsuka, Kanagawa 254-0014 Japan.*

[b] *NISSAN ARC, LTD. 1, Natsushima-cho, Yokosuka, Kanagawa 237-0061 Japan.*

[c] *School of Chemisrty, The University of Edinburgh. Joseph Black Building, David Brewster Road, Edinburgh EH9 1FJ, The U.K.*

*Email: orito.yuya.k6@daiichisankyo.co.jp

Table of contents

XAS instruments setup and scanning conditions ... 2
- Fluorescence mode XAS measurement ... 2
- Transmission XAS measurement ... 2
- XAS data processing, LCAs ... 2

Materials ... 2

Reference samples preparation ... 2

Experimental setup and typical procedures ... 4
- Fluorescence mode XAS measurement ... 4
- Background evaluations using water in photometry cells ... 4
- In situ reaction monitoring of Pd/C with HCl by transmission XAS ... 4
- HCl slow addition (Figure 8) ... 5
- HCl intermittent addition (Figure 9) ... 5
- LCA for in situ reaction monitoring ... 6

Supplemental experimental results ... 9
- Background level of whole scan area ... 9
- Initial investigation of the reaction of Pd/C with HCl (fluorescence mode XAS, Figure 6) ... 9
- The reactions of Pd/C with other acids (Fluorescence mode XAS) ... 10
- The reaction of Pd/C with HCl and analysis of liquid and solid phase after separation (Figure 10) ... 10
- The temperature dependency of the amount of Pd leaching into solution ... 11

References ... 11

XAS instruments setup and scanning conditions

XAS measurements were carried out using PF-AR NW10A beamline of the Photon Factory (PF) in the High Energy Accelerator Research Organization (KEK, Tsukuba, Japan). The storage ring was operated at 6.5 GeV. The white light was monochromatized using a water-cooled double crystal monochromator equipped with Si(311) crystal. The XAS spectra of the Pd K-edge (24.35 keV) were collected in transmission and fluorescence modes. The X-ray beam size is 2 mm (wide) x 1 mm (hight) by using 4-quadrant slit. The incident X-ray beam intensity (I0) were monitored by a 170 mm-long ionization chamber with Ar gas 100 %.

Fluorescence mode XAS measurement

The X-ray fluorescence was detected by a 19-element germanium solid state detector (Ortec, USA). The sample was located vertically at 45° of the incident X-ray direction. The measurement time was 20 minutes for 1 spectrum, and the energy range of the measurement is from 24020 eV to 25140 eV. For performing EXAFS analysis, background parameters as follows: pre-edge range is from -300 to -60 eV at E_0 (a fraction of the edge step); Normalization range is from 150 to 550 eV; Rbkg is 1.0. Fourier transformation parameters as follows: k-weight is 2; k-range is from 2 to 10 $Å^{-1}$; with Hanning window function.

Transmission XAS measurement

The transmitted X-ray beam intensity were monitored by ionization chamber with Ar gas 50 % and Kr gas 50%. The measurement time was 1 minute for 1 spectrum, and the energy range of the measurement is from 24200 eV to 24700 eV. For performing EXAFS analysis, background parameters as follows: pre-edge range is from -350 to -150 eV at E_0 (a fraction of the edge step); Normalization range is from 100 to 1450 eV; Rbkg is 1.0. Fourier transformation parameters as follows: k-weight is 2; k-range is from 2 to 14 $Å^{-1}$; with Hanning window function.

XAS data processing, LCAs

XAS data were processed by IFEFFIT and Athena software. LCA was performed using data from appropriate reference sample for each data processing, and the fitting range is from -20 eV to 30 eV at E_0.

Materials

Pd/C was purchased from Kawaken Fine Chemicals (Type M, 5 wt%, ca. 50% wet) and stored under ambient atmosphere unless otherwise noted. Water was processed with Millipore Milli-Q. Hydrochloric acid and other acids are purchased from Wako Pure Chemicals and used as received. Reference $[Pd^{II}Cl_4]^{2-}$ solution was prepared from Na_2PdCl_4 purchased from Sigma-Aldrich. For $PdCl_2$, in-house standard in KEK was used as prepared. Spectrophotometry cells made of PS and PMMA, reaction vessels made of PP and PFA were purchased from As One corporation.

Reference samples preparation

To perform LCAs accurately for Pd/C, in-house standard of Pd^0 (foil) nor $Pd^{II}O$ (powder) were not appropriate, possibly due to difference of crystallinity and particle size of metal, those common standard sample gave slightly

different spectra to reference samples prepared from nanoparticle Pd/C (Figure S1 for example). Reduced Pd^0/C was prepared by treatment of hydrogen gas (1 atm) for 2 hours under room temperature, and sealed in glass ampule under N_2 atmosphere. Oxide enriched PdO/C was prepared following literature procedure[1] and stored under ambient atmosphere.

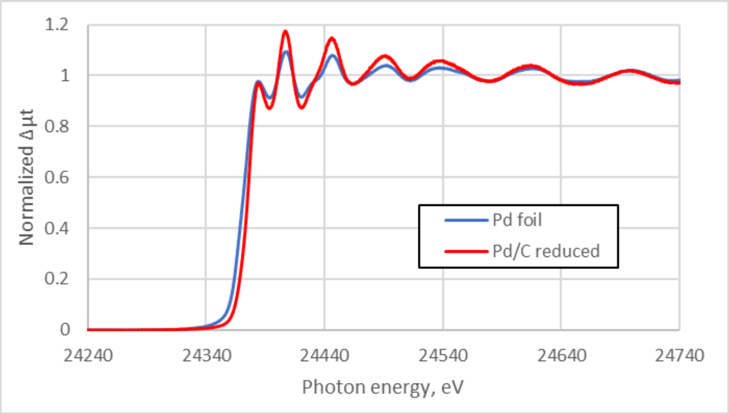

Figure S1. XANES obtained from Pd foil and reduced Pd/C.

The Pd/C used for leaching experiments consisted of various lots (#). Oxidation states determined by LCA for each lot of Pd/C are shown in Table S1.

Pd/C	Lot #	Pd^0, %	$Pd^{II}O$, %	Storage condition
A	#5676	0	100	>1 year in shelf
B	#5690	6.8	93.2	3 weeks after shipping from vendor
C	#5690	6.4	93.6	3 weeks after shipping from vendor

Table S1. Oxidation states of Pd/C.

Experimental setup and typical procedures

Fluorescence mode XAS measurement

To a glass HSGC vial (from Agilent technology, crimp cap: Cat. No. 5183-4474, screw cap: Cat. No. 5188-2753), added were sample and magnetic stirrer bar (when needed) then the vial was sealed. X-ray beam was irradiated at the side of vial, about 5 mm depth from the wall. Fluorescence mode XAS was recorded until satisfactory S/N ratio obtained (typically 20 min).

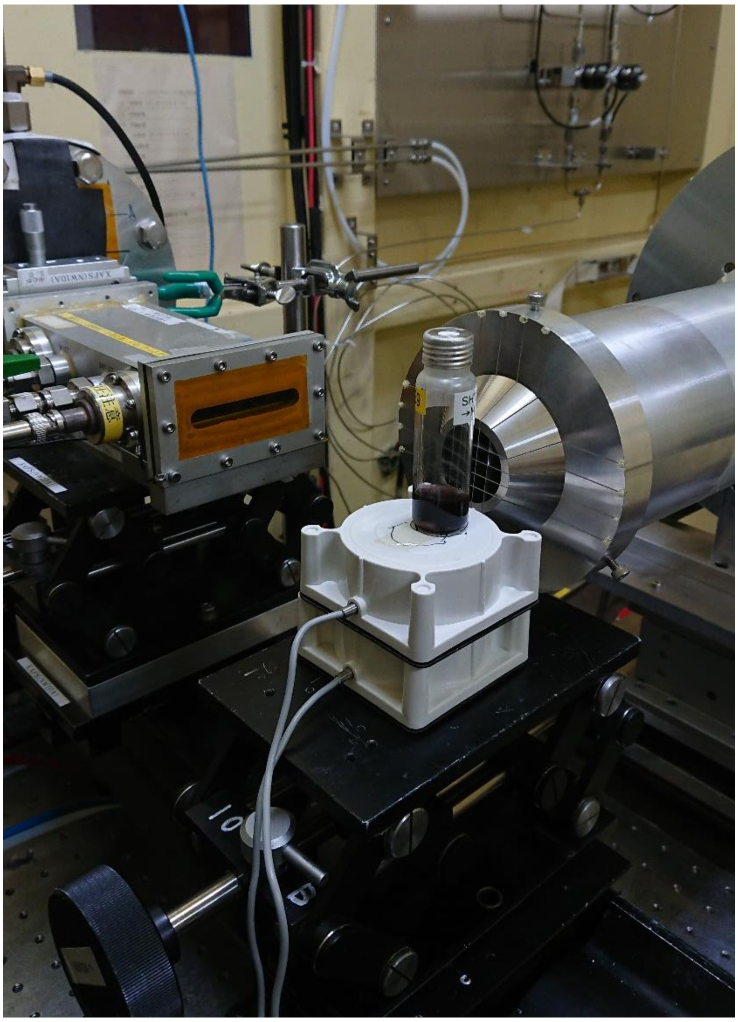

Figure S2. Typical setup for fluorescence mode XAS measurement.

Background evaluations using water in photometry cells

To a rectangular spectrophotometer cell with 1 mm wall thickness added was water and the cell was sealed by lid. The cell was placed on the beamline and μt was measured by transmission XAS.

In situ reaction monitoring of Pd/C with HCl by transmission XAS

To achieve a short scan interval, the scan range was narrowed to 24200~24700 eV for reaction monitoring experiments.

HCl slow addition (Figure 8)

To a 200 mL PP cylindrical bottle added magnetic stirrer bar, Pd/C (50 g as mixture of lot A 25 g and lot B 25 g, contains ca. 1.25 g as of Pd^0, 11.7 mmol of Pd species in total, ca. 0.72% Pd concentration in gross slurry), and water (146 mL) then the bottle was sealed by a silicone stopper fitted with bimetal thermometer probe and PFA tube connected to syringe pump. The mixture was stirred on the Radleys Carousel stirring hotplate in 800 rpm, maintaining the temperature at around 25ºC. Then conc. HCl (6.9 mL, 82.8 mmol) was added dropwise to the slurry over a period of 30 minutes via syringe pump. The X-ray beam irradiation was centred in the lower middle section of the bottle to avoid interference from the vortex arising from mixing. The reaction progress was monitored by transmission XAS at 2 minute intervals.

HCl intermittent addition (Figure 9)

To a 200 mL PP cylindrical bottle was added a magnetic stirrer bar, Pd/C (50 g of lot C, contains ca. 1.25 g of Pd^0, 11.7 mmol of Pd species in total, ca. 0.72% Pd concentration in gross slurry), and water (149 mL) then the bottle was sealed by silicone stopper fitted with bimetal thermometer probe and PFA tube connected to syringe pump. The mixture was stirred on the Radleys Carousel stirring hotplate in 800 rpm, maintaining temperature around 25ºC. Then conc. HCl (4.1 mL, 49.7 mmol) was added to the slurry in 3 portions, with 10 minutes intervals between each addition, via syringe pump. The X-ray beam irradiation was centred in the lower middle section of the bottle to avoid interference from the vortex arising from mixing. The reaction progress was monitored by transmission XAS at 2 minute intervals.

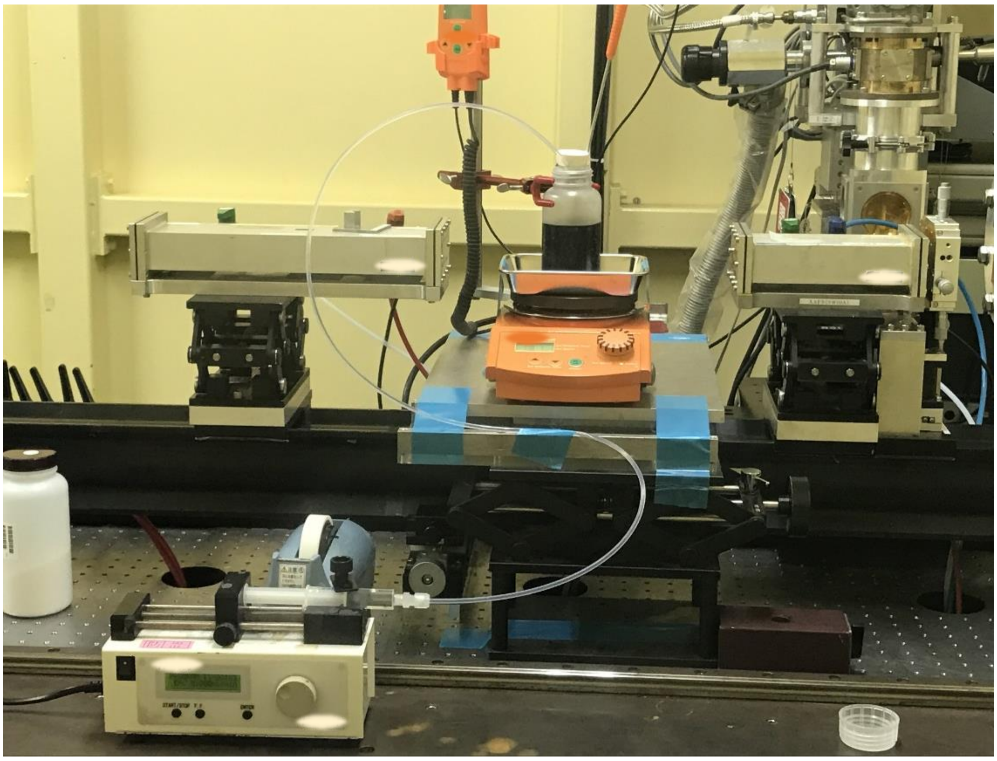

Figure S3. Experiment setup for in situ transmission XAS measurement for Pd/C slurry, using PP bottle.

LCA for in situ reaction monitoring

2 component LCA was performed using spectra of the initial and final mixtures. Data are shown in Tables S2 and S3. For HCl slow addition (Figure 8), the chart is drawn using data based on 5 point moving-average to decrease interference of noise as mentioned in the main article.

	LCA results		Moving average 5pts	
Scans	Scan #0	Scan #54	Initial	Final
1	1	0	1	0
2	1	0	1	0
3	1	0	1	0
4	1	0	1	0
5	1	0	0.994	0.006
6	1	0	0.9838	0.0162
7	0.97	0.03	0.964	0.036
8	0.949	0.051	0.941	0.059
9	0.901	0.099	0.9018	0.0982
10	0.885	0.115	0.864	0.136
11	0.804	0.196	0.8214	0.1786
12	0.781	0.219	0.7726	0.2274
13	0.736	0.264	0.7158	0.2842
14	0.657	0.343	0.6722	0.3278
15	0.601	0.399	0.6154	0.3846
16	0.586	0.414	0.5702	0.4298
17	0.497	0.503	0.542	0.458
18	0.51	0.49	0.5144	0.4856
19	0.516	0.484	0.4936	0.5064
20	0.463	0.537	0.4898	0.5102
21	0.482	0.518	0.4876	0.5124
22	0.478	0.522	0.48	0.52
23	0.499	0.501	0.4748	0.5252
24	0.478	0.522	0.4696	0.5304
25	0.437	0.563	0.4296	0.5704
26	0.456	0.544	0.3884	0.6116
27	0.278	0.722	0.3462	0.6538
28	0.293	0.707	0.318	0.682
29	0.267	0.733	0.2798	0.7202
30	0.296	0.704	0.2786	0.7214
31	0.265	0.735	0.283	0.717
32	0.272	0.728	0.2974	0.7026

33	0.315	0.685	0.326	0.674
34	0.339	0.661	0.3486	0.6514
35	0.439	0.561	0.3612	0.6388
36	0.378	0.622	0.351	0.649
37	0.335	0.665	0.3354	0.6646
38	0.264	0.736	0.3026	0.6974
39	0.261	0.739	0.2802	0.7198
40	0.275	0.725	0.2574	0.7426
41	0.266	0.734	0.2528	0.7472
42	0.221	0.779	0.246	0.754
43	0.241	0.759	0.2414	0.7586
44	0.227	0.773	0.1998	0.8002
45	0.252	0.748	0.1638	0.8362
46	0.058	0.942	0.126	0.874
47	0.041	0.959	0.0902	0.9098
48	0.052	0.948	0.0412	0.9588
49	0.048	0.952	0.0402	0.9598
50	0.007	0.993	0.035	0.965
51	0.053	0.947	0.033	0.967
52	0.015	0.985	0.02925	0.97075
53	0.042	0.958	0.036667	0.963333

Table S2. LCA results and calculated moving average from HCl slow addition monitoring experiment.

	LCA results	
Scans	Scan #0	Scan #42
1	0.969	0.031
2	0.95	0.05
3	0.929	0.071
4	0.71	0.29
5	0.696	0.304
6	0.718	0.282
7	0.675	0.325
8	0.692	0.308
10[a]	0.678	0.322
11	0.657	0.343
12	0.665	0.335
13	0.408	0.592
14	0.295	0.705

15	0.282	0.718
16	0.241	0.759
17	0.245	0.755
18	0.209	0.791
19	0.207	0.793
20	0.187	0.813
21	0.206	0.794
22	0.221	0.779
23	0.158	0.842
24	0.149	0.851
25	0.104	0.896
26	0.121	0.879
27	0.088	0.912
28	0.052	0.948
29	0.048	0.952
30	0.053	0.947
31	0.057	0.943
32	0.051	0.949
33	0.08	0.92
34	0.059	0.941
35	0.015	0.985
36	0.045	0.955
37	0.045	0.955
38	0.031	0.969
39	0	1
40	0	1
41	0.008	0.992

[a] Scan #9 was omitted due to noise.

Table S3. LCA results from HCl intermittent addition monitoring experiment.

Supplemental experimental results

Background level of whole scan area

With PP and PFA vessels containing water, transmission XAS was measured and ut was recorded between (24000 ev to 25900 ev) for various optical pathlength, Figure S5.

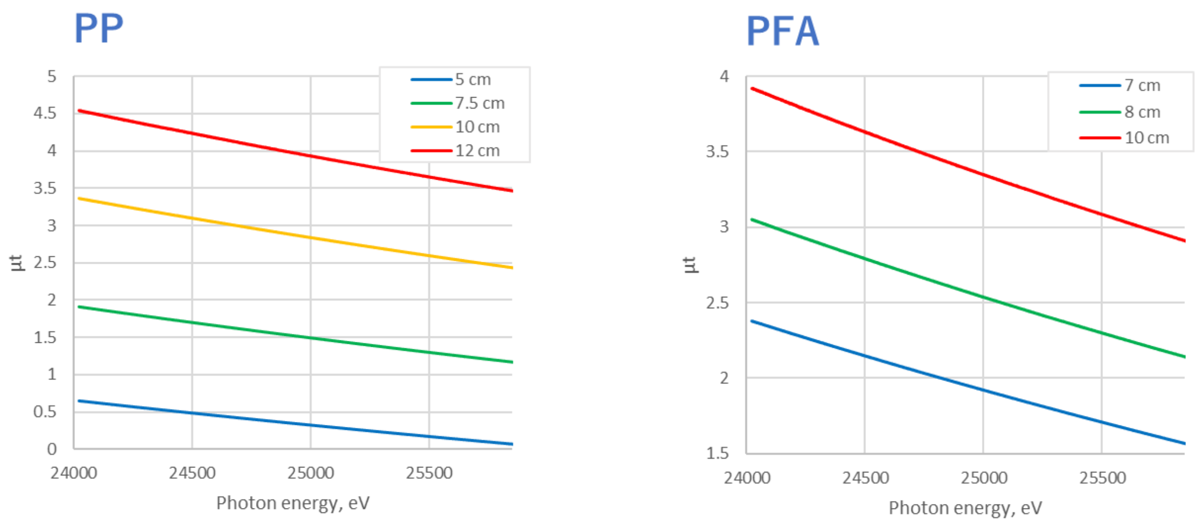

Figure S4. Background level for whole scan range.

Initial investigation of the reaction of Pd/C with HCl (fluorescence mode XAS, Figure 6)

To a screw top glass HSGC vial (from Agilent technology, Cat. No. 5188-5392), was added a magnetic stirrer bar, Pd/C (100 mg), and HCl (3 N, 4 mL). The vial was then sealed and stirred for 2 hours at room temperature. The vial was then stored for 1 week and fluorescence mode XAS was measured, with stirring, on the beamline. X-ray beam was irradiated at the side of vial, about 5 mm depth from the wall. Fluorescence mode XAS was recorded until a satisfactory S/N ratio obtained (about 20 min). The resulting data were compared with spectra of initial Pd/C (slurry in MeOH) and an in-house standard sample of $PdCl_2$.

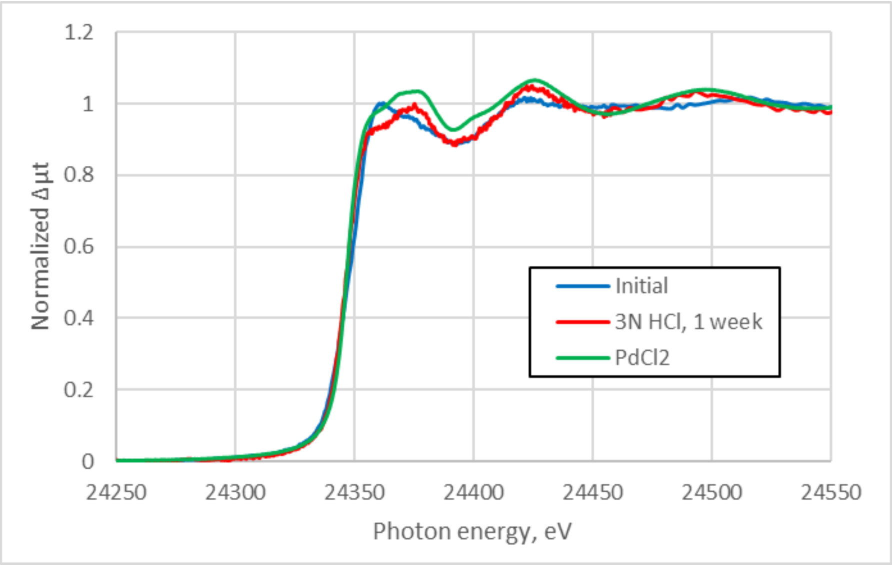

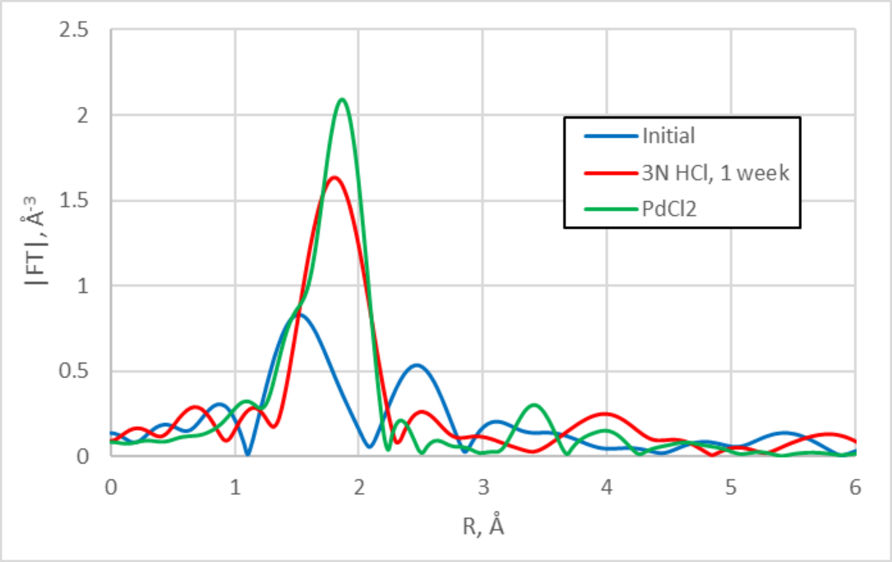

Figure S5. XANES and EXAFS spectra after HCl treatment.

The reactions of Pd/C with other acids (Fluorescence mode XAS)

To a screw top glass HSGC vial (from Agilent technology, Cat. No. 5188-5392), was added a magnetic stirrer bar, Pd/C (100 mg), and acid (3 N, 4 mL). The vial was then sealed and stirred for 2 hours at room temperature. The vial was then stored for 1 week and fluorescence mode XAS was measured, with stirring, on the beamline. X-ray beam was irradiated at the side of vial, about 5 mm depth from the wall. Fluorescence mode XAS was recorded until a satisfactory S/N ratio obtained (about 20 min).

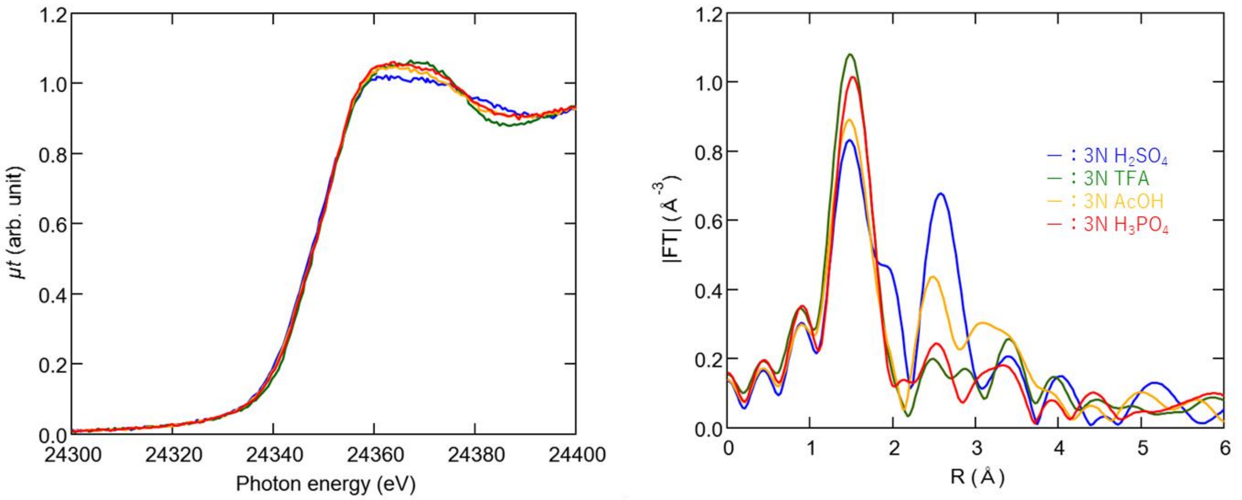

Figure S6. The reactions of Pd/C with various acids.

The reaction of Pd/C with HCl and analysis of liquid and solid phase after separation (Figure 10)

To a 100 mL round-bottomed flask fitted with magnetic stirrer bar, was added Pd/C (10.0 g) and aq. HCl (1 N, 100 mL) then the mixture was stirred for 0.5 h at room temperature. The slurry was then filtered and washed with water. The resulting solids were sealed in an ampoule under a N_2 atmosphere and measured by XAS. The collected eluent liquid was diluted up to 200 mL using a measuring flask, and 1 mL of resulting liquid sampled and diluted up to 200

mL (corresponds to 40,000 mL dilution) to be measured by Zeeman atomic absorption spectrometer (ThermoFisher iCE3400, 21.5 ppm for eluent liquid, 4.3 mg as Pd metal). Since the initial Pd/C (5%, 50% wet) contains ca. 250 mg Pd, the amount of leaching into liquid phase was calculated to be 2% of total Pd.

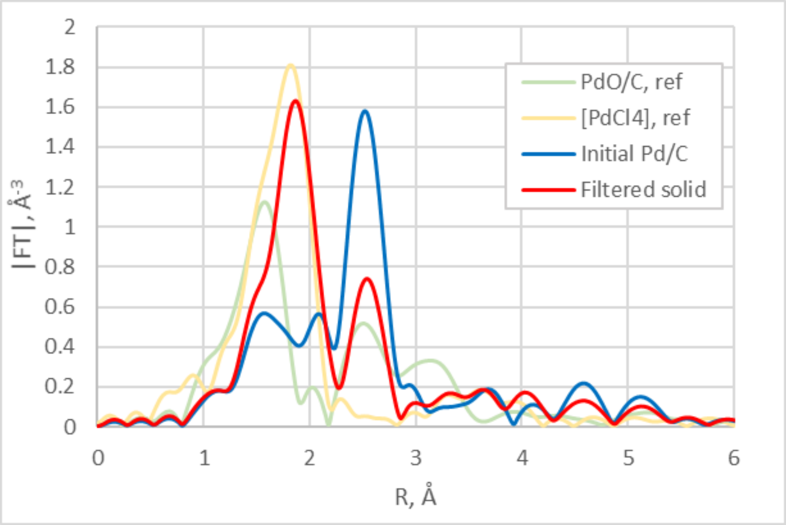

Figure S7. EXAFS data of the mixed-state Pd/C before and after HCl treatment, with reference samples.

The temperature dependency of the amount of Pd leaching into solution

To a 150 mL glass reaction vessel fitted teflon impeller, was added conc. HCl (4.86 g), water (53.1 mL), and MeOH (53.1 mL) then stirred at target temperature in Eyela ChemStation (PPV-5460). Into the mixture Pd/C (Kawaken Type M, 5% Pd, 50% wet, 1.0 g) was added and resulting slurry was maintained at the same temperature. 1.0 mL of the liquid phase was sampled through syringe filter for each sample, diluted 10,000 times, and the Pd concentration determined by Zeeman atomic absorption spectrometer (ThermoFisher iCE3400).

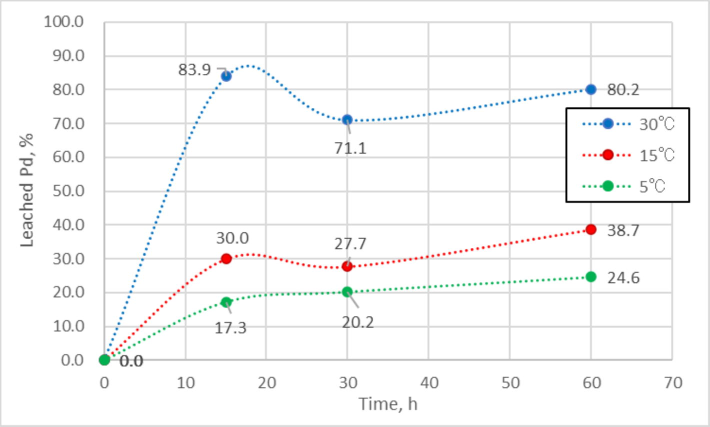

Figure S8. Pd leaching amount in solution phase under various temperature conditions.

References

(1) Rossy, C.; Majimel, J.; Delapierre, M. T.; Fouquet, E.; Felpin, F.-X. On the Peculiar Recycling Properties of Charcoal-Supported Palladium Oxide Nanoparticles in Sonogashira Reactions. *Appl Catal Gen* **2014**, *482*, 157–162. https://doi.org/10.1016/j.apcata.2014.05.019.
